# A predictive map learned from diverse entorhinal inputs explains the role of context-dependent reorganization of hippocampal place cells

**DOI:** 10.3389/fncom.2026.1780000

**Published:** 2026-05-11

**Authors:** Yusuke Kuniyoshi, Tadashi Yamazaki

**Affiliations:** Graduate School of Informatics and Engineering, The University of Electro-Communications, Tokyo, Japan

**Keywords:** hippocampus, lateral entorhinal cortex, medial entorhinal cortex, place cell, predictive map, reinforcement learning, sparse coding

## Abstract

The hippocampus is thought to support spatial memory and navigation by constructing predictive representations of the environment. Predictive map theory formalizes this function as a successor representation (SR). However, existing models assume a fixed and uniform distribution of place fields, despite experimental findings that place cell density is dynamically modulated by rewards and objects. Here, we propose a biologically inspired neural model in which predictive maps emerge from diverse entorhinal inputs. In the model, place cell-like representations are generated via non-negative sparse coding of medial entorhinal spatial signals and lateral entorhinal contextual and motivational signals, and are subsequently transformed into predictive maps using successor features. By coupling the predictive map to an actor–critic framework, the model supports goal-directed navigation in continuous environments. Furthermore, the model reproduces experience-dependent restructuring of hippocampal representations, including object-centered overrepresentation of place fields in two-dimensional environments and reward-centered overrepresentation in one-dimensional environments. Together, these results demonstrate that hippocampal predictive maps can emerge from the integration of diverse entorhinal inputs, providing a unified account of how spatial, contextual, and motivational information jointly shape hippocampal representations and behavior.

## 1 Introduction

Spatial memory and navigation are fundamental cognitive functions that enable animals to understand their environment and adapt their behavior. These functions are primarily supported by the hippocampus and the entorhinal cortex (EC). Among these regions, the hippocampus contains place cells that fire selectively when the animal is at specific locations, known as place fields (O'Keefe and Dostrovsky, 1971). Each place cell has a distinct preferred location, and the ensemble activity of the population collectively encodes the animal's current location (O'Keefe, 1976). This ensemble activity is thought to constitute an internal map of space (O'Keefe and Nadel, 1978). The EC provides diverse spatial and contextual inputs to the hippocampus. In the medial entorhinal cortex (MEC), grid cells exhibit periodic firing fields arranged in a hexagonal lattice (Hafting et al., 2005), while boundary cells encode proximity to environmental borders (Solstad et al., 2008). In contrast, the lateral entorhinal cortex (LEC) is thought to convey task-relevant and non-spatial information, such as rewards and objects (Issa et al., 2023; Deshmukh and Knierim, 2011). Through the integration of these heterogeneous inputs from the EC, hippocampal place cells are thought to construct a unified representation of space that incorporates geometric structure, environmental features, and motivational significance.

Experimental studies have demonstrated that place cells encode not only the animal's current location but also future trajectories (Mehta et al., 2000; Alvernhe et al., 2011). To explain the predictive nature of place cell activity, Stachenfeld et al. (2017) proposed that the hippocampus functions as a predictive map, formalized as the successor representation (SR) (Dayan, 1993), inspired by reinforcement learning (RL) theory. The SR represents how often each state is expected to be visited in the future, starting from a given state, thereby capturing the predictive structure of the environment. The predictive map theory has provided a unified framework for interpreting various experimental findings that were difficult to explain previously. Recent studies have explored biologically plausible implementations of predictive maps in the hippocampal circuit (Fang et al., 2023; Bono et al., 2023; George et al., 2023). Notably, George et al. (2023) demonstrated that a combination of theta phase precession (O'Keefe and Recce, 1993) and spike-timing dependent plasticity (STDP) (Bi and Poo, 1998), both physiologically observed in the hippocampus, can approximate the SR from the activity of hippocampal CA3 place cells in continuous environments. Their model makes a significant contribution by theoretically linking STDP and theta phase precession—two mechanisms that had previously been considered independently of SR learning.

However, existing models of hippocampal predictive maps assume that the spatial locations of place cells are predefined (Fang et al., 2023; Bono et al., 2023; George et al., 2023). In these models, the SR is learned over a fixed set of place fields, implicitly assuming a uniform and stable spatial tiling of the environment. In contrast, experimental studies have shown that hippocampal place cells are not necessarily distributed uniformly across space (Gauthier and Tank, 2018; Vandrey et al., 2021). The place-field density can be dynamically modulated by contextual factors, such as rewards or salient objects, leading to overrepresentation of behaviorally relevant locations. These observations suggest that predictive maps in the hippocampus should not be learned over a predetermined set of place fields. Rather, they should emerge from the integration of diverse inputs from the EC, including spatial signals from the MEC and contextual or motivational signals from the LEC. Accordingly, a model that constructs predictive maps from the EC inputs, rather than assuming fixed place fields, may provide a more realistic account of hippocampal function. Such a framework may also help elucidate the functional roles of distinct EC cell types within a reinforcement learning context.

In this study, we propose a biologically inspired neural model that learns SR-like predictive maps from diverse inputs in the EC. Our model consists of two stages. First, hippocampal place cell-like representations are generated by a recurrent network that computes non-negative sparse coding (NSC) from entorhinal inputs. Second, these representations are transformed into predictive representations using successor features. This unified framework reproduces the skew of place fields (Mehta et al., 2000). Building on this predictive representation, we further demonstrate that the model can support goal-directed behavior. By coupling the predictive map to an actor-critic framework, the agent is able to estimate rewards and compute state values, enabling spatial navigation in continuous environments. Crucially, our model does not assume a fixed spatial organization of place fields. Instead, by incorporating context-dependent signals from the LEC, it captures experience-dependent restructuring of hippocampal representations. In a two-dimensional environment containing objects, the model similarly reproduces object-centered overrepresentation of place cells (Vandrey et al., 2021). In a one-dimensional environment with rewards, the model reproduces the overrepresentation of place cells near a reward location (Gauthier and Tank, 2018). Together, these results demonstrate that predictive maps can emerge from diverse EC inputs and may provide a unifying account of how spatial, contextual, and motivational information shape hippocampal representations.

Preliminary results were presented in a conference paper (Kuniyoshi and Yamazaki, 2025).

## 2 Methods

### 2.1 Overview of the model architecture

**Figure 1:**
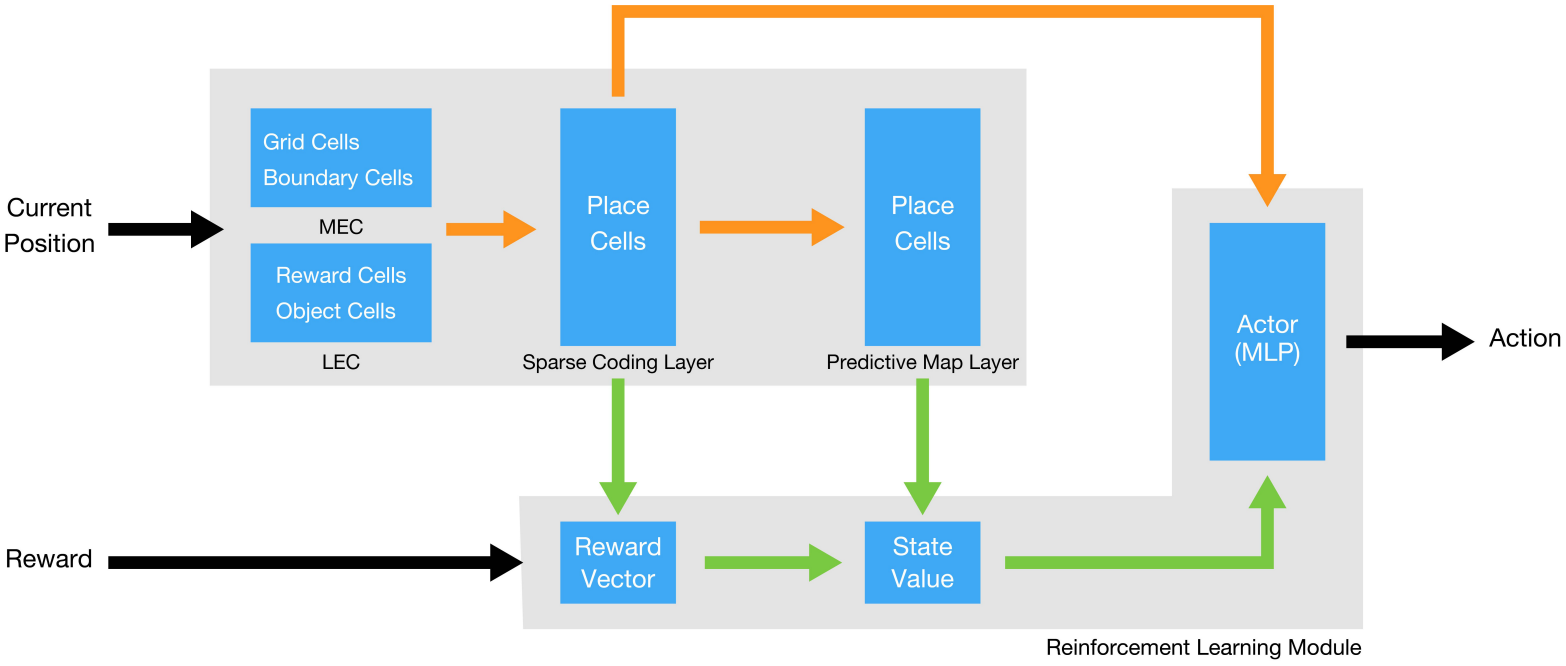
Overall architecture of the model. Orange arrows indicate synaptic connections, green arrows represent pathways related to the RL module, and black arrows denote the overall input and output.

The proposed model consists of two major components: a biologically inspired neural network that simulates the entorhinal-hippocampal circuit, and an RL module that enables the agent to learn in a continuous environment (Figure 1). The role of the neural network is to compute a predictive map of space from entorhinal inputs, which is subsequently used by the RL module to learn value functions and action policies.

The neural network contains three layers: an entorhinal input layer, a sparse coding layer, and a predictive map layer. The input layer consists of four types of entorhinal signals: (1) grid cell activity from the MEC, (2) boundary-tuned input activity that conveys proximity to periodic environmental borders (implemented only in two-dimensional environments), (3) object-related activity from the LEC (implemented only in two-dimensional environments), and (4) reward-related activity from the LEC (implemented only in one-dimensional environments). In tasks without explicit reward or object cues, only the MEC-derived spatial signals (grid cells and boundary-tuned input) are provided. The sparse coding layer receives these entorhinal signals and generates place-cell-like activity patterns. The predictive map layer then receives the place-cell activity and computes a predictive representation, such as the SR. These two layers may correspond to distinct hippocampal subregions, such as CA3 and CA1, respectively.

The output of the sparse coding and predictive map layers is used to compute a reward vector, a value function, and an action policy within the RL module. Each computational component and its underlying mechanisms are described in the following sections.

### 2.2 A model of the entorhinal cortex

#### 2.2.1 Grid cells in the medial entorhinal cortex

**Figure 2:**
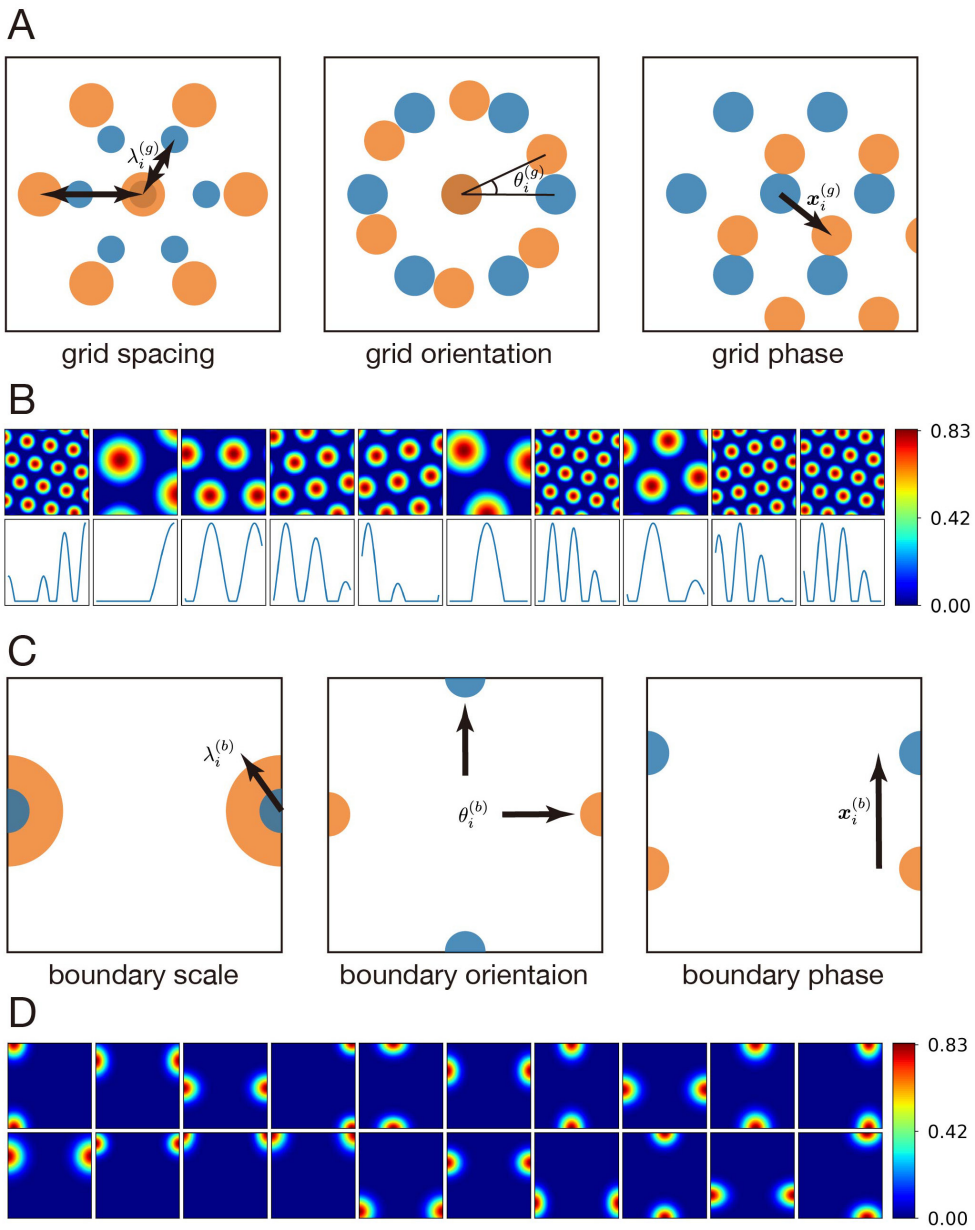
Neuronal activities in the MEC. **(A)** The activity of MEC grid cells is uniquely determined by three parameters: grid spacing ($\lambda_{i}^{\left(g\right)}$), grid orientation ($\theta_{i}^{\left(g\right)}$), and grid phase ($x_{i}^{\left(g\right)}$). **(B)** Top: Examples of grid cell activity in a two-dimensional environment (1 m × 1 m). Bottom: Examples of grid cell activity in a one-dimensional environment (1 m). **(C)** The activity of MEC boundary-tuned inputs is uniquely determined by three parameters: boundary scale ($\lambda_{i}^{\left(b\right)}$), boundary orientation ($\theta_{i}^{\left(b\right)}$), and boundary phase ($x_{i}^{\left(b\right)}$). **(D)** Examples of boundary-tuned input activity in a two-dimensional environment (1 m × 1 m).

Grid cells in the MEC exhibit hexagonal spatial firing fields that provide spatial input to the hippocampus. In our model, these grid-cell inputs are combined with both boundary-tuned MEC inputs (in two-dimensional environments) and object- or reward-related inputs from the LEC, described in the following subsections. To implement grid cells in the MEC, we took the same approach as in Solstad et al. (2006); Lian and Burkitt (2021). Specifically, in a two-dimensional environment (1 m × 1 m), the firing activity *g*_*i*_(***x***) of the *i*-th grid cell at the agent's current location ***x*** = (*x, y*) is defined as follows (Equations 1 and 2):


$$\begin{array}{l} g_{i}\left(x\right)=\frac{2}{3}\left(\frac{1}{3}\overset{3}{\underset{j=1}{\sum }}\cos\left(\frac{4\pi }{\sqrt{3}\lambda_{i}^{\left(g\right)}}u_{j}\cdot \left(x-x_{i}^{\left(g\right)}\right)\right)\right), \end{array}$$ (1)

$$\begin{array}{l} u_{j}=\left(\cos\left(\frac{2\pi j}{3}+\theta_{i}^{\left(g\right)}\right),\sin\left(\frac{2\pi j}{3}+\theta_{i}^{\left(g\right)}\right)\right), \end{array}$$ (2)

where $\lambda_{i}^{\left(g\right)}$ is the grid spacing, $\theta_{i}^{\left(g\right)}$ is the grid orientation, $x_{i}^{\left(g\right)}=\left(x_{i},y_{i}\right)$ is the grid phase, ***u***_*j*_ is the unit vector with direction $2\pi j/3+\theta_{i}^{\left(g\right)}$. The firing activity of a grid cell is determined by three independent parameters: grid spacing, grid orientation, and grid phase (Figure 2A).

Following the previous work (Lian and Burkitt, 2021), we used the same set of biologically plausible parameters based on experimental observations in rats (Figure 2B). The minimum grid spacing $\lambda_{i}^{\left(g\right)}$ was set to 28 cm, consistent with experimental data (Hafting et al., 2005), and scaled by a factor of 1.42 (Stensola et al., 2012) to obtain four spacings, all under 1 m (28, 39.76, 56.46, and 80.17 cm). To introduce diversity, six grid orientations were used: 0, 60, 120, 180, 240, and 300°. The grid phase ***x***_*i*_ was uniformly selected from five equally spaced positions within the interval $\left[0,\lambda_{i}^{\left(g\right)}\right]$. The total number of grid cells *N*_g_ was the product of the number of spacings, orientations, and phases, resulting in *N*_g_ = 4 × 6 × 25 = 600.

The activity vector of the *N*_g_ grid cells at the agent's location ***x***_*t*_ at time *t* is denoted by ***s***_g, *t*_ and defined as follows (Equation 3):


$$\begin{array}{l} s_{\text{g},t}={\left(g_{1}\left(x_{t}\right),\cdots \;,g_{N_{\text{g}}}\left(x_{t}\right)\right)}^{⊤} \end{array}$$ (3)

In addition to two-dimensional environments, our model also considers one-dimensional environments. Pröll et al. (2018) demonstrated that grid-cell activity on a linear track can be interpreted as a one-dimensional slice of the two-dimensional hexagonal firing pattern as long as the animal moves in a consistent direction. This suggests that the underlying two-dimensional representation remains intact even in one-dimensional settings.

Motivated by this finding, we defined one-dimensional grid-cell activity by constraining the animal's *y*-coordinate to a constant value *y*_0_ and evaluating the two-dimensional grid-cell response along the line ***x*** = (*x, y*_0_). Specifically, the activity of the *i*-th grid cell in a one-dimensional environment is given by (Equation 4):


$$\begin{array}{l} g_{i}\left(x\right)=g_{i}\left(\left(x,y_{0}\right)\right). \end{array}$$ (4)

In this formulation, the spacing, orientation, and phase parameters remain identical to the two-dimensional case, ensuring that the one-dimensional firing fields arise naturally as a restriction of the same underlying hexagonal lattice (Figure 2B). This approach enables a unified treatment of grid-cell coding in both one- and two-dimensional environments without introducing additional parameters (Figure 2B).

#### 2.2.2 Boundary-tuned inputs in the medial entorhinal cortex

In two-dimensional environments, we additionally introduced boundary-tuned inputs that encode spatial signals associated with the coordinate boundaries of the environment. These inputs are not intended to represent biologically identified entorhinal cell types such as border cells (Solstad et al., 2008), but rather serve as auxiliary inputs to provide additional spatial cues.

In our simulations, periodic boundary conditions are imposed, meaning that opposite edges of the environment are treated as topologically equivalent (i.e., forming a two-dimensional torus). Although grid cell activity is inherently periodic, representing it within a bounded coordinate system introduces discontinuities at the boundaries. As a result, spatially adjacent locations across boundaries produce dissimilar activity patterns, leading to inconsistent spatial representations.

The boundary-tuned inputs were therefore introduced to mitigate this representational discontinuity by informing the agent of the locations where these discontinuities occur. Consequently, these inputs should be interpreted as a modeling device rather than direct analogs of biological neurons. The firing activity *b*_*i*_(***x***) of the *i*-th boundary-tuned input at the agent's current location ***x*** = (*x, y*) is defined as follows (Equation 5):


$$b_{i}(x)=\{\begin{array}{ll} 0.83\cdot \exp\text{​}\left(-\frac{\min{\left(x,L-x\right)}^{2}}{2{\left(\lambda_{i}^{\left(b\right)}\right)}^{2}}\right)\exp\text{​}\left(-\frac{{\left(y-y_{i}^{\left(b\right)}\right)}^{2}}{2{\left(\lambda_{i}^{\left(b\right)}\right)}^{2}}\right) & \text{if }\theta_{\text{i}}^{\left(\text{b}\right)}\text{ = 0},\\ 0.83\cdot \exp\text{​}\left(-\frac{\min{\left(y,L-y\right)}^{2}}{2{\left(\lambda_{i}^{\left(b\right)}\right)}^{2}}\right)\exp\text{​}\left(-\frac{{\left(x-x_{i}^{\left(b\right)}\right)}^{2}}{2{\left(\lambda_{i}^{\left(b\right)}\right)}^{2}}\right) & \text{if }\theta_{\text{i}}^{\left(\text{b}\right)}\text{=1}, \end{array}$$ (5)

where the boundary scale $\lambda_{i}^{\left(b\right)}$ represents the size of the spatial receptive field. The boundary orientation $\theta_{i}^{\left(b\right)}$ indicates whether the input responds to left-right boundaries ($\theta_{i}^{\left(b\right)}=0$) or top-bottom boundaries ($\theta_{i}^{\left(b\right)}=1$). The boundary phase $x_{i}^{\left(b\right)}=\left(x_{i}^{\left(b\right)},y_{i}^{\left(b\right)}\right)$ represents the preferred location along the boundary direction (Figure 2C). *L* denotes the size of the two-dimensional environment, and the min(*x, L*−*x*) or min(*y, L*−*y*) computes the shortest distance to the nearest boundary (Figure 2D).

The total number of boundary-tuned inputs was *N*_b_ = 300. The parameters $\lambda_{i}^{\left(b\right)}$ and $x_{i}^{\left(b\right)}$ were uniformly sampled from the ranges [0.08 m, 0.12 m] and [0 m, *L* m], respectively. The boundary orientation $\theta_{i}^{\left(b\right)}$ was assigned such that half of the inputs responded to left-right boundaries ($\theta_{i}^{\left(b\right)}=0$) and the other half responded to top-bottom boundaries ($\theta_{i}^{\left(b\right)}=1$). The constant 0.83 was introduced to match the peak activity of boundary-tuned inputs to that of grid cells. Finally, the activity vector of all boundary-tuned inputs at time *t* is denoted as (Equation 6):


$$\begin{array}{l} s_{\text{b},t}={\left(b_{1}\left(x_{t}\right),\cdots \;,b_{N_{\text{b}}}\left(x_{t}\right)\right)}^{⊤}. \end{array}$$ (6)

By combining the activity of the grid cells and boundary-tuned inputs, the overall spatial representation in the MEC is expressed as (Equation 7):


$$\begin{array}{l} s_{\text{m},t}=\left(\begin{array}{c} s_{\text{g},t}\\ s_{\text{b},t} \end{array}\right), \end{array}$$ (7)

which serves as the input to the sparse coding layer. In one-dimensional environments, boundary-tuned inputs are not used. Therefore, the overall spatial representation in the MEC is expressed as ***s***_m, *t*_ = ***s***_g, *t*_.

#### 2.2.3 Object cell activity in the lateral entorhinal cortex

**Figure 3:**
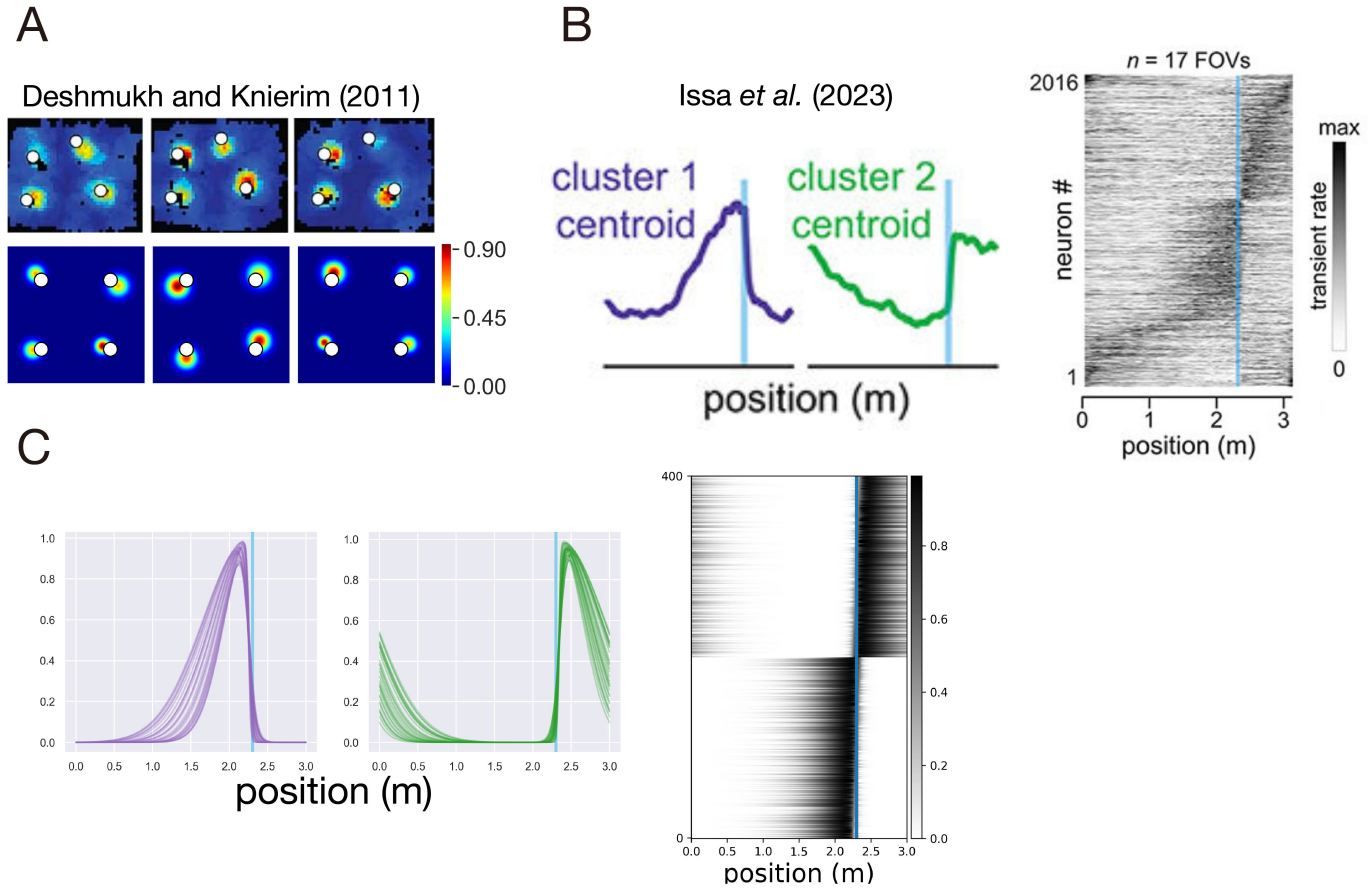
Neuronal activities in the LEC. **(A)** Receptive fields of LEC object cells. Top: experimentally recorded receptive fields of object cells in the LEC, adapted from the bioRxiv preprint by Issa et al. (2023). Bottom: receptive fields of LEC object cells generated by the proposed model. **(B)** Experimentally recorded receptive fields of reward-related neurons in the LEC, including pre-reward and post-reward cells, adapted from the bioRxiv preprint by Issa et al. (2023). **(C)** Modeled receptive fields of pre-reward and post-reward cells in a one-dimensional environment from Deshmukh and Knierim (2011).

Deshmukh and Knierim (2011) reported that a subset of neurons in the LEC, called object cells, exhibit spatially localized firing fields anchored to objects placed in the environment. Specifically, these object cells respond strongly when the animal approaches an object and show reduced activity at distant locations (Figure 3A). Importantly, Vandrey et al. (2021) demonstrated that the presence of objects in environments increases the proportion of hippocampal CA1 place cells that fire near those objects. These findings suggest that object-related information encoded by LEC object cells may contribute to the overrepresentation of place cells firing in proximity to objects.

Motivated by these experimental findings, we modeled LEC object cells in a two-dimensional environment (1 m × 1 m). We placed four objects at fixed locations [(0.25 m, 0.25 m), (0.25 m, 0.75 m), (0.75 m, 0.25 m), (0.75 m, 0.75 m)] and each object cell was assigned a preferred firing center near one of these object locations. The center ***c***_*i, j*_ of the *i*-th object cell for the *j*-th object location ***o***_*j*_ was sampled as (Equation 8):


$$\begin{array}{l} c_{i,j}=o_{j}+\epsilon_{i,j},\;\epsilon_{i,j}\sim U\left(-0.07\text{ m},0.07\text{ m}\right), \end{array}$$ (8)

where ***ϵ***_*i, j*_ denotes the perturbation around the *j*-th object location and *U*(*a, b*) is a uniform distribution from *a* to *b*. Thus, each object cell has one preferred location for each of the four objects, producing a set of object-anchored firing fields.

The firing activity of the *i*-th object cell at the location ***x*** is defined as the sum of Gaussian fields centered on each object (Equation 9):


$$\begin{array}{l} o_{i}\left(x\right)=\overset{4}{\underset{j=1}{\sum }}\alpha_{i,j}\exp\left(-\frac{||x-c_{i,j}|{|}^{2}}{2\sigma_{i,j}^{2}}\right), \end{array}$$ (9)

where the scale parameter α_*i, j*_ and the size of the receptive-field σ_*i, j*_ are independently sampled as α_*i, j*_~*U*(0.5 m, 1.0 m), σ_*i, j*_~*U*(0.03 m, 0.06 m) (Figure 3A).

Let *N*_o_ = 600 denote the total number of LEC object cells. At the agent's location ***x***_*t*_, the activity vector of the object cells is defined as follows (Equation 10):


$$\begin{array}{l} s_{\text{o},t}={\left(o_{1}\left(x_{t}\right),\cdots \;,o_{N_{\text{o}}}\left(x_{t}\right)\right)}^{⊤}. \end{array}$$ (10)

Although the present study relies on allocentric representation (Deshmukh and Knierim, 2011), recent work has also reported that some LEC neurons encode object locations in egocentric coordinates and exhibit direction-selective tuning (Wang et al., 2018).

#### 2.2.4 Reward cell activity in the lateral entorhinal cortex

Issa et al. (2023) demonstrated that, in a linear-track reward-learning task, distinct neuronal populations in the LEC exhibit selective activity immediately before or after the reward location (Figure 3B). These pre-reward and post-reward cells are thought to provide context-dependent signals related to reward expectation and reward consumption.

In parallel, Gauthier and Tank (2018) has shown that reward learning induces an overrepresentation of hippocampal place fields near reward locations in one-dimensional environments. Reward-modulated signals in the LEC are thought to contribute to the reward-centered overrepresentation of hippocampal place fields observed in reward-learning tasks.

Motivated by these experimental findings, we introduced two types of reward-modulated inputs in a one-dimensional environment: (1) pre-reward cells that become active immediately before the reward, and (2) post-reward cells that become active immediately after the reward. Specifically, the firing activity *r*_*i*_(*x*) of the *i*-th pre-reward or post-reward cell at the location *x* in the one-dimensional environment is defined as follows (Equations 11-13):


$$\begin{array}{l} r_{i}\left(x\right)=\exp\left(-\frac{{\left(x-\left(r+\delta_{i}\right)\right)}^{2}}{2\sigma_{i}^{2}}\right)\cdot G_{i}\left(x\right), \end{array}$$ (11)

$$G_{i}(x)=\{\begin{array}{l} 1-S\text{​}\left(\frac{x-(r+\delta_{i})}{\Delta_{i}}\right)\text{(pre-reward cell)},\\ 1-S\text{​}\left(\frac{(r+\delta_{i})-x}{\Delta_{i}}\right)\text{(post-reward cell)}, \end{array}$$ (12)

$$\begin{array}{l} S\left(x\right)=\frac{1}{1+e^{-x}}, \end{array}$$ (13)

where δ_*i*_~*U*(−0.05 m, 0.05 m) is the offset from the reward location, σ_*i*_~*U*(0.3 m, 0.6 m) is the size of the receptive field, Δ_*i*_~*U*(0.01 m, 0.05 m) is the sharpness of the activity transition around the reward location, *G*_*i*_(*x*) is the gate function, and *S*(*x*) is the sigmoid function. The firing activity of a pre-reward and post-reward cell is determined by three independent parameters (Figure 3C).

The activity vector of the *N*_r_ reward-modulated cells (*N*_r_ = 600: 300 pre-reward and 300 post-reward cells) at the agent's current location *x*_*t*_ in the one-dimensional environment is defined as follows (Equation 14):


$$\begin{array}{l} s_{\text{r},t}={\left(r_{1}\left(x_{t}\right),\cdots \;,r_{N_{\text{r}}}\left(x_{t}\right)\right)}^{⊤}. \end{array}$$ (14)

Although the present study adopts the pre-reward and post-reward cell types (Issa et al., 2023), recent work has also reported that reward-related information in the LEC may be represented in a spatially structured manner (Bowler and Losonczy, 2023).

### 2.3 Sparse coding layer

The sparse coding layer receives neuronal activity from the MEC and the LEC and generates place cell activity using the NSC model proposed by Lian and Burkitt (2021). This model generates localized and sparse place-cell-like activity from grid-cell inputs. The non-negative constraint reflects two biological considerations: neuronal firing rates are inherently non-negative, and projections from MEC to hippocampal pyramidal neurons are predominantly excitatory. Moreover, Dong et al. (2021) reported that backward skew is prominent in CA1 place cells but largely absent in CA3 place cells; because the Lian and Burkitt model produces CA3-like place-cell activity patterns, we adopted it as the sparse coding layer.

The NSC minimizes the following cost function (Equations 15 and 16):


$$\begin{array}{l} G\left(A,s_{\text{h}}\right)=||s_{\text{e}}-As_{\text{h}}|{|}_{2}^{2}+\beta ||s_{\text{h}}|{|}_{1}, \end{array}$$ (15)

$$\begin{array}{l} s_{\text{e},t}=\left(\begin{array}{c} s_{\text{m},t}\\ s_{\text{r},t}\\ s_{\text{o},t} \end{array}\right), \end{array}$$ (16)

where ***s***_e_ is the entorhinal input vector, *A* is an *N*_e_×*N*_h_ basis matrix, *N*_e_ = *N*_m_+*N*_r_+*N*_o_ is the number of neurons in the EC, *N*_h_ is the number of neurons in the sparse coding layer, ***s***_h_ is the output vector representing neuronal activities in the sparse coding layer, and β is a sparsity-regularization parameter. The terms ||·||_1_ and ||·||_2_ denote the *L*_1_ and *L*_2_ norms, respectively. The first term represents reconstruction error, while the second term penalizes large values in ***s***_h_. Non-negativity constraints are imposed on both *A* and ***s***_h_.

To minimize Equation 15, the local competitive algorithm (LCA) (Rozell et al., 2008), which can be implemented as a biologically inspired dynamical system, was adopted in a discretized manner (Equations 17 and 18):


$$\begin{array}{l} u_{\text{h},t+1}=u_{\text{h},t}+\frac{1}{\tau }\left(-u_{\text{h},t}+A_{t}^{⊤}s_{\text{e},t}-W_{t}s_{\text{h},t}\right), \end{array}$$ (17)

$$\begin{array}{l} s_{\text{h},t+1}=\max\left(u_{\text{h},t+1}-\beta ,0\right), \end{array}$$ (18)

where τ = 10 is a time constant, and $W_{t}=A_{t}^{⊤}A_{t}-I$ represents recurrent inhibition among hippocampal neurons. The sparse coding layer is learned on a faster time scale than the predictive map layer. As a result, the sparse representation allows the predictive map layer to learn from relatively stable inputs.

The basis matrix *A*_*t*_ is updated via the following Hebbian-like learning rule (Equations 19 and 20):


$$\begin{array}{l} {Ã}_{t+1}=A_{t}+\eta \left(s_{\text{e},t}-A_{t}s_{\text{h},t}\right)s_{\text{h},t}^{⊤}, \end{array}$$ (19)

$$\begin{array}{l} A_{t+1}=\max\left({Ã}_{t+1},0\right), \end{array}$$ (20)

where η is the learning rate. After each update, each column of *A* is normalized to have unit *L*_2_ norm.

### 2.4 Predictive map layer

The predictive map layer receives neuronal activity ***s***_h_ from the sparse coding layer and computes a predictive representation ***p***_h_ as follows (Equation 21),


$$\begin{array}{l} p_{\text{h},t}=M_{t}^{⊤}s_{\text{h},t}, \end{array}$$ (21)

where *M*_*t*_ is an *N*_h_×*N*_h_ connection matrix at time *t*. This computation corresponds to estimating the expected future occupancy of states represented by the sparse coding layer. The connection matrix *M*_*t*_ is updated via the following TD-based learning rule (Equation 22):


$$\begin{array}{l} M_{t+1}=M_{t}+\eta s_{\text{h},t}{\left[s_{\text{h},t}+\gamma p_{\text{h},t+1}-p_{\text{h},t}\right]}^{⊤}, \end{array}$$ (22)

where η and γ denote the learning rate and the discount factor, respectively.

The proposed formulation is closely related to the SR framework (Dayan, 1993; Stachenfeld et al., 2017). In classical SR formulations, the environment is discretized into a finite set of states, and the SR matrix $M_{\text{SR}}\left(s,s^{\prime }\right)$ is defined as follows (Equation 23):


$$\begin{array}{l} M_{\text{SR}}\left(s,s^{\prime }\right)=E\left[\overset{\infty }{\underset{t=0}{\sum }}\gamma^{t}I\left(s_{t}=s^{\prime }\right)|s_{0}=s\right], \end{array}$$ (23)

where *I*(·) denotes the indicator function. The SR encodes the discounted future occupancy of state *s*′ starting from state *s*.

More generally, the successor features (SF) framework extends this formulation to continuous state representations (de Cothi and Barry, 2020; Bennett et al., 2025). Let ***ϕ***(*s*) denote a feature representation of state *s*. The successor feature vector is defined as follows (Equation 24):


$$\begin{array}{l} \psi \left(s\right)=E\left[\overset{\infty }{\underset{t=0}{\sum }}\gamma^{t}\phi \left(s_{t}\right)|s_{0}=s\right]. \end{array}$$ (24)

In the present model, the sparse coding activity ***s***_h, *t*_ plays the role of the feature representation ***ϕ***(*s*), and the predictive map activity ***p***_h, *t*_ corresponds to the successor feature vector ***ψ***(*s*). Thus, the predictive map layer can be interpreted as computing successor features over the distributed state representation generated by the sparse coding layer.

### 2.5 Reinforcement learning module

#### 2.5.1 Reward estimation

Within the RL module, immediate reward signals are mapped onto the place-cell-like activity in the sparse coding layer, yielding an estimate of the immediate reward vector defined over place-field centers. This reward vector is updated according to the following rule:


$$\begin{array}{l} R_{t+1}=R_{t}+\alpha \left[r\left(x_{t}\right)s_{\text{h},t}-R_{t}\right], \end{array}$$ (25)

where *R*_*t*_ denotes the current estimate of the reward vector, *r*(***x***_*t*_) is the scalar reward obtained at the agent's location ***x***_*t*_, and ***s***_h, *t*_ represents the activity of the sparse coding layer. The parameter α controls the learning rate. This update rule incrementally adjusts *R*_*t*_ toward the reward-weighted place-cell activity *r*(***x***_*t*_)***s***_h, *t*_, thereby enabling the association between spatial representations and rewards. Notably, because the update is performed online, the reward estimate can smoothly adapt when the reward location is changed, reflecting newly experienced reward contingencies.

#### 2.5.2 State value computation

Using the estimated reward vector *R*_*t*_ obtained from Equation 25, the state value at the agent's location ***x***_*t*_ is computed as


$$\begin{array}{l} V\left(x_{t}\right)=p_{\text{h},t}^{⊤}R_{t}, \end{array}$$ (26)

where ***p***_h, *t*_ denotes the neuronal activity in the predictive map layer. Because this layer encodes expected future state occupancy in continuous spatial locations, the resulting state value naturally extends to continuous spatial locations.

#### 2.5.3 Policy learning

The policy determines the agent's action based on the activity of the sparse coding layer. Two types of policies were used depending on the dimensionality of the environment: a one-dimensional Gaussian policy and a two-dimensional population-vector policy. Both policies were trained under the same actor-critic framework, in which the actor generates actions and the critic evaluates the state value computed in Equation 26, and the actor is implemented as a multi-layer perceptron (MLP). The policy parameters were optimized by policy-gradient learning using the TD error (Equations 27 and 28):


$$\begin{array}{l} E_{\text{loss}}=-\log\pi \left(d_{t}|x_{t}\right)\delta_{t}, \end{array}$$ (27)

$$\begin{array}{l} \delta_{t}=r\left(x_{t}\right)+\gamma V\left(x_{t+1}\right)-V\left(x_{t}\right), \end{array}$$ (28)

where π denotes the policy, and δ_*t*_ is the TD error.

##### 2.5.3.1 Two-dimensional policy

In the two-dimensional environment, the actor was implemented as a population-vector decoder to maintain biological plausibility. The MLP of the actor produced 32 directional weights corresponding to evenly spaced movement directions θ_*k*_∈[0, 2π). Specifically, the actor consisted of a two-layer MLP with a LayerNorm input, followed by a sigmoid-weighted linear unit activation, and a softmax output (Equation 29):


$$\begin{array}{l} w_{t}=f_{\phi }\left(s_{\text{h},t}\right), \end{array}$$ (29)

where ***w***_*t*_ = (*w*_*t*, 1_, ⋯ , *w*_*t*, 32_) represents the normalized directional weights and *f*_ϕ_(·) represents the MLP. The mean movement direction **μ**_*t*_ = (μ_*t, x*_, μ_*t, y*_) was computed as a population vector (Equation 30):


$$\begin{array}{l} \mu_{t}=d_{\text{max}}\overset{32}{\underset{k=1}{\sum }}w_{t,k}\left(\cos\theta_{k},\sin\theta_{k}\right), \end{array}$$ (30)

where *d*_max_ = 0.1 m represents the maximum step size. The actual displacements Δ***x***_*t*_ = (Δ*x*_*t*_, Δ*y*_*t*_) were then sampled from Gaussian policies over the *x*- and *y*-axes (Equation 31):


$$\begin{array}{l} \Delta x_{t}\sim N\left(\mu_{t,x},\sigma^{2}\right),\;\Delta y_{t}\sim N\left(\mu_{t,y},\sigma^{2}\right), \end{array}$$ (31)

where σ = 0.02 m. To ensure bounded movements, the sampled displacements were clipped to the range [−2*d*_max_ m, 2*d*_max_ m]. This design allows the actor to represent smooth directional preferences, analogous to motor cortical neurons that encode movement direction via population vectors (Georgopoulos et al., 1982).

##### 2.5.3.2 One-dimensional policy

In the one-dimensional environment, the actor was implemented as a simple Gaussian policy that determined the agent's displacement Δ*x*_*t*_ along the *x*-axis. Specifically, the output layer of the MLP is the policy mean μ_*t*_ (Equation 32):


$$\begin{array}{l} \mu_{t}=f_{\phi }\left(s_{\text{h},t}\right), \end{array}$$ (32)

where *f*_ϕ_(·) represents the MLP. The MLP used LayerNorm, a rectified linear unit activation function, and a tanh output unit, ensuring that the action value was bounded between −1 and 1. The output μ_*t*_ was then rescaled to the range [*d*_min_ m, *d*_max_ m], where *d*_min_ = 0.01 m and *d*_max_ = 0.1 m defined the maximum movement step. The final action was sampled from a Gaussian distribution centered at μ_*t*_ (Equation 33):


$$\begin{array}{l} \Delta x_{t}\sim N\left(\mu_{t},\sigma^{2}\right), \end{array}$$ (33)

with a fixed standard deviation σ = 0.01 m. To ensure bounded movements, the minimum displacement was clipped to *d*_min_ m. This simple Gaussian policy was sufficient for the one-dimensional task, where the agent's movement was restricted to a single spatial dimension.

## 3 Results

### 3.1 Constructing predictive maps and state value functions from MEC inputs

To examine whether the proposed model can construct predictive maps from MEC inputs, we simulated an agent navigating a periodic two-dimensional environment of size 1 m × 1 m under a fixed policy. The agent predominantly moved upward at a constant speed of 10 cm per step, while its head direction was perturbed by small angular noise sampled from a standard normal distribution. We introduced an upward bias to examine whether the place fields exhibit a downward skew. However, the specific direction of the bias was not essential.

**Figure 4:**
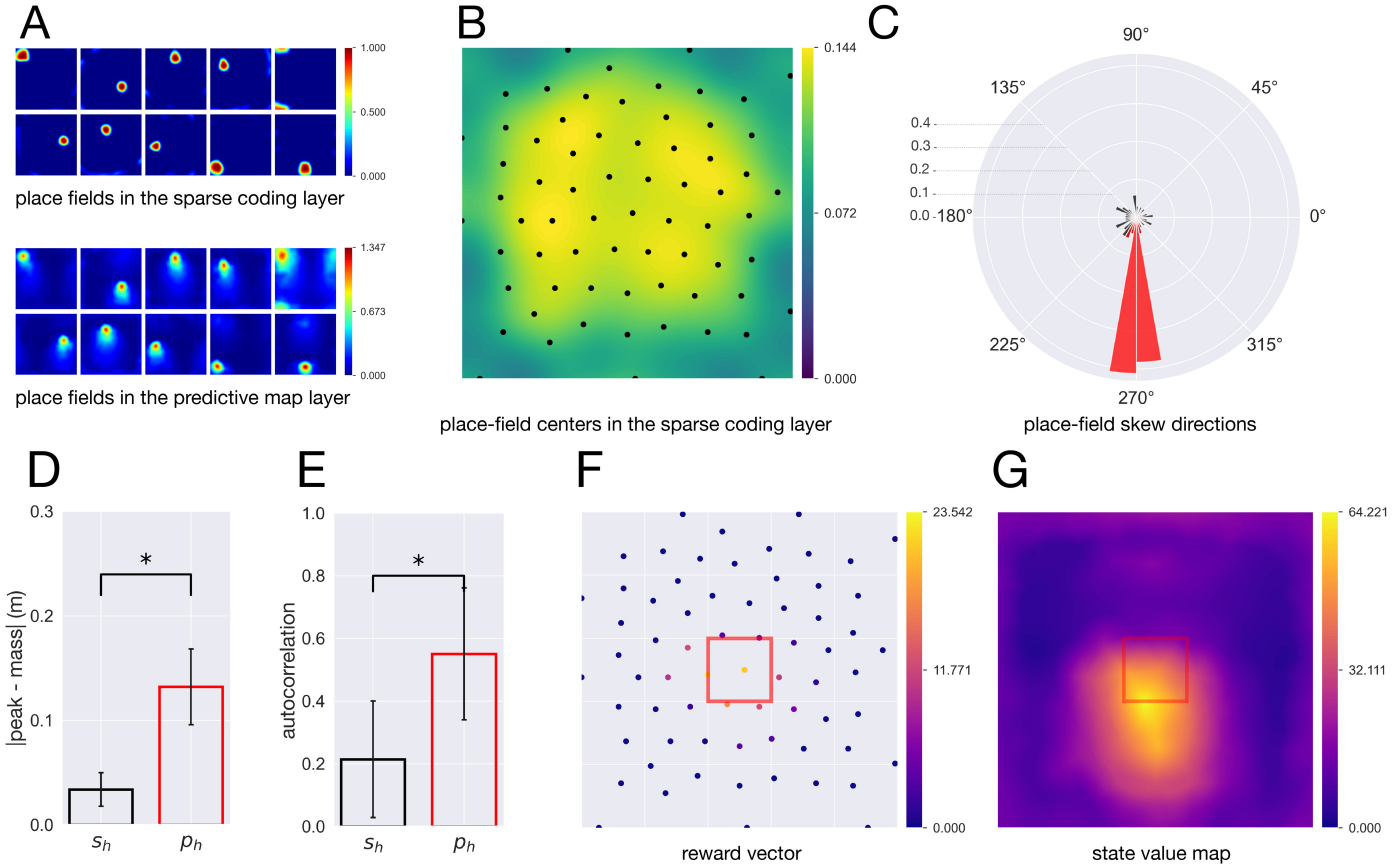
Comparison of place-cell properties between the sparse coding and predictive map layers. **(A)** Top: 10 representative place fields in the sparse coding layer. Bottom: 10 representative place fields in the predictive map layer. **(B)** Spatial distribution of place-field centers in the sparse coding layer. Dots indicate the peak activity locations of each place field, and the background shows the kernel density estimate (KDE) of their spatial distribution. **(C)** Rose plots of place-field skew directions. Black: sparse coding layer; red: predictive map layer. **(D)** Length of the place-field skew vectors. Bars represent the mean ± SD across neurons. Black: sparse coding layer; red: predictive map layer. **(E)** Lag-1 autocorrelation of neuronal activity over time. Bars represent the mean ± SD across neurons. Black: sparse coding layer; red: predictive map layer. **(F)** Learned reward vector after reward learning. Each point corresponds to a spatial location, and red indicates the reward location. **(G)** State value function. Red indicates the reward location.

Neuronal activities in the sparse coding layer formed place fields distributed across the environment (Figure 4A), consistent with the previous work (Lian and Burkitt, 2021). These place-field centers covered the entire environment (Figure 4B). Although experimental studies report enrichment of place fields near environmental boundaries (e.g., Tanni et al., 2022), the simulations use periodic boundary conditions rather than physical walls. Therefore, the spatial distribution of place fields is determined by the learned sparse representation and does not necessarily reproduce boundary-related enrichment. Whereas the sparse coding layer showed symmetric place fields, the predictive map layer exhibited place fields that were systematically skewed (Figure 4A). Because the agent predominantly moved upward, the place fields were skewed downward, reflecting predictive locations along the experienced trajectory. This phenomenon can be regarded as a two-dimensional analog of the backward skew of place fields observed on linear tracks in experimental studies (Mehta et al., 2000), suggesting that the predictive map layer encodes predictive information.

To characterize the predictive nature of each layer, we performed a quantitative comparison of neuronal activity patterns in the sparse coding and predictive map layers using two complementary measures. As the first measure, we quantified spatial skew as the vector from the peak activity location to the center of mass of each place field. The resulting vectors were uniformly distributed in the sparse coding layer, whereas those in the predictive map layer showed a pronounced downward bias (Figure 4C). In addition, the magnitude of this vector was substantially larger in the predictive map layer than in the sparse coding layer ( 0.034 ± 0.016 m vs. 0.132 ± 0.036 m; Welch's *t*-test, *p* < 0.01; Figure 4D).

As a second quantitative measure, we evaluated the temporal consistency of neuronal activity by computing the lag-1 autocorrelation. Neuronal activity in the predictive map layer exhibited significantly higher autocorrelation than that in the sparse coding layer ( 0.551 ± 0.210 vs. 0.214 ± 0.186; Welch's *t*-test, *p* < 0.01; Figure 4E). Taken together with the spatial skew analysis, these findings indicate that the predictive map layer captures future-oriented structure in neural activity, whereas the sparse coding layer predominantly reflects instantaneous spatial representations.

We then examined whether the predictive map layer could support the computation of a state value function. To this end, a reward of 10 was delivered when the agent entered a central region of the environment defined by 0.4 ≤ *x* ≤ 0.6 and 0.4 ≤ *y* ≤ 0.6.

During learning, the estimated reward vector selectively increased for spatial locations associated with reward delivery (Figure 4F). Notably, the learned reward values did not exactly coincide with the true reward magnitude. This difference can be attributed to the learning rule, in which reward estimates are updated only when neurons in the sparse coding layer are active within the reward region, causing the estimated reward vector to depend on the agent's visitation frequency.

The state value function was subsequently computed as the inner product between the learned reward vector and neuronal activity in the predictive map layer (Figure 4G). The resulting value map exhibited elevated values not only at the reward location itself but also in regions located below the reward area. These results demonstrate that, beyond encoding predictive spatial information, the predictive map layer enables value computation when combined with a simple reward learning mechanism.

### 3.2 The predictive map layer is essential for solving the water-maze task

To examine the functional importance of the predictive map, we applied the proposed model to a reinforcement learning setting based on an Actor–Critic framework. In this framework, the Actor was implemented as a MLP that received neuronal activity from the sparse coding layer as the state representation and produced continuous displacements along the *x*- and *y*-axes.

Simulations of 1,000 episodes were performed in the same two-dimensional environment and reward configuration as described in the previous section. At the beginning of each episode, the agent was initialized at a random location near the boundary of the environment: either the *x*- or *y*-coordinate was sampled from an edge interval ([0.0, 0.1] or [0.9, 1.0]), while the remaining coordinate was drawn uniformly from [0.0, 1.0]. Episodes ended when the agent successfully reached the reward region.

**Figure 5:**
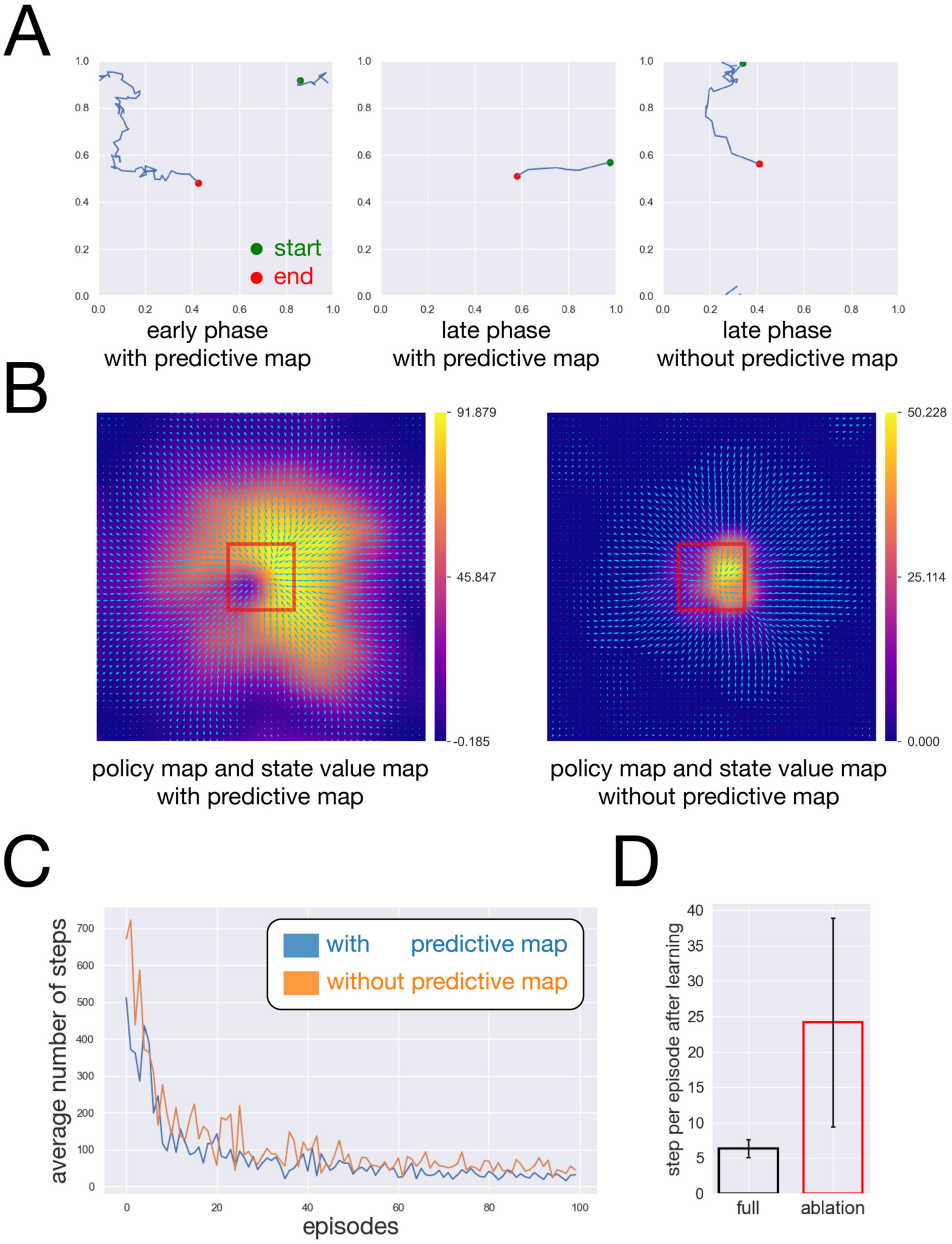
Effect of the predictive map layer on policy learning and navigation performance. **(A)** Agent trajectories in different phases of learning. Left: trajectory (green: start, red: end) during the early phase (first 100 episodes). Middle: trajectory during the late phase (last 100 episodes). Right: trajectory during the late phase after ablation of the predictive map layer. Each simulation consisted of 1,000 episodes in total. **(B)** Learned policy map (arrows) and state value function (color map). Left: model with the predictive map layer; Right: model without the predictive map layer. **(C)** Learning curves showing the average number of steps required to reach the reward region over 100 episodes. Results are averaged over 10 independent runs with different random initial conditions. Blue: model with the predictive map layer; orange: model without the predictive map layer. **(D)** Distribution of the number of steps required to reach the reward region over the final 100 episodes. Black: model with the predictive map layer; red: model without the predictive map layer.

Agent trajectories during different phases of learning are shown in Figure 5A. During the early phase (first 100 episodes), the agent exhibited largely exploratory behavior. After sufficient learning, trajectories during the late phase (last 100 episodes) showed more directed navigation toward the reward location. Following sufficient learning, the agent successfully navigated toward the reward region, and the learned policy and state value function guided movement toward the reward location (Figure 5B).

Learning performance was evaluated by tracking the number of steps required for the agent to reach the reward over 100 episodes. This analysis revealed a progressive reduction in the number of steps over learning (Figure 5C), indicating the successful acquisition of goal-directed navigation behavior. These findings suggest that predictive representations provided by the predictive map layer effectively support reinforcement learning in the water-maze task.

To directly assess the contribution of the predictive map layer, we conducted an ablation experiment in which the state value function was computed using neuronal activity from the sparse coding layer instead of the predictive map layer, while all other components and training protocols were kept unchanged.

Without the predictive map layer, the learned policy displayed poorly directed behavior in regions distant from the reward (Figure 5A). Because the sparse coding layer represents only the agent's instantaneous location and does not encode predictive information, the corresponding state value function failed to propagate across space. As a result, effective policy learning was largely restricted to areas near the reward, leading to inefficient navigation from more distant locations (Figure 5B).

Quantitative comparison revealed that the ablated model required a larger number of steps to reach the reward and showed slower convergence across episodes (Figures 5C, D). Together, these results demonstrate that the predictive map layer plays a critical functional role in enabling efficient learning of goal-directed behavior in the water-maze task. The slight difference in performance during the first episode (Figure 5C) is attributed to stochastic variability in the exploration process rather than an inherent structural advantage of the predictive map layer prior to learning.

### 3.3 Object-related modulation of place cells by LEC object cells

Deshmukh and Knierim (2011) demonstrated that, in two-dimensional environments containing objects, a subset of neurons in the LEC exhibits spatially localized firing fields near those objects. Subsequently, Vandrey et al. (2021) reported that the proportion of place cells with object-centered place fields increases in the distal CA1, which receives significant inputs from the LEC.

In the present study, we modeled LEC object cells as reported by Deshmukh and Knierim (2011) and incorporated them into the input to the sparse coding layer, in addition to grid cells and boundary-tuned inputs from the MEC. Using this extended input representation, we aimed to reproduce the experimental finding of Vandrey et al. (2021), namely the increased proportion of place cells with receptive fields near objects.

For the simulation, we introduced a two-dimensional environment, in which four objects were placed at fixed locations [(0.25, 0.25), (0.25, 0.75), (0.75, 0.25), and (0.75, 0.75)]. The agent freely explored the environment by random movement. With such simulation settings, we compared the spatial distribution of place cells between two conditions: with and without LEC object cells included in the model input.

**Figure 6:**
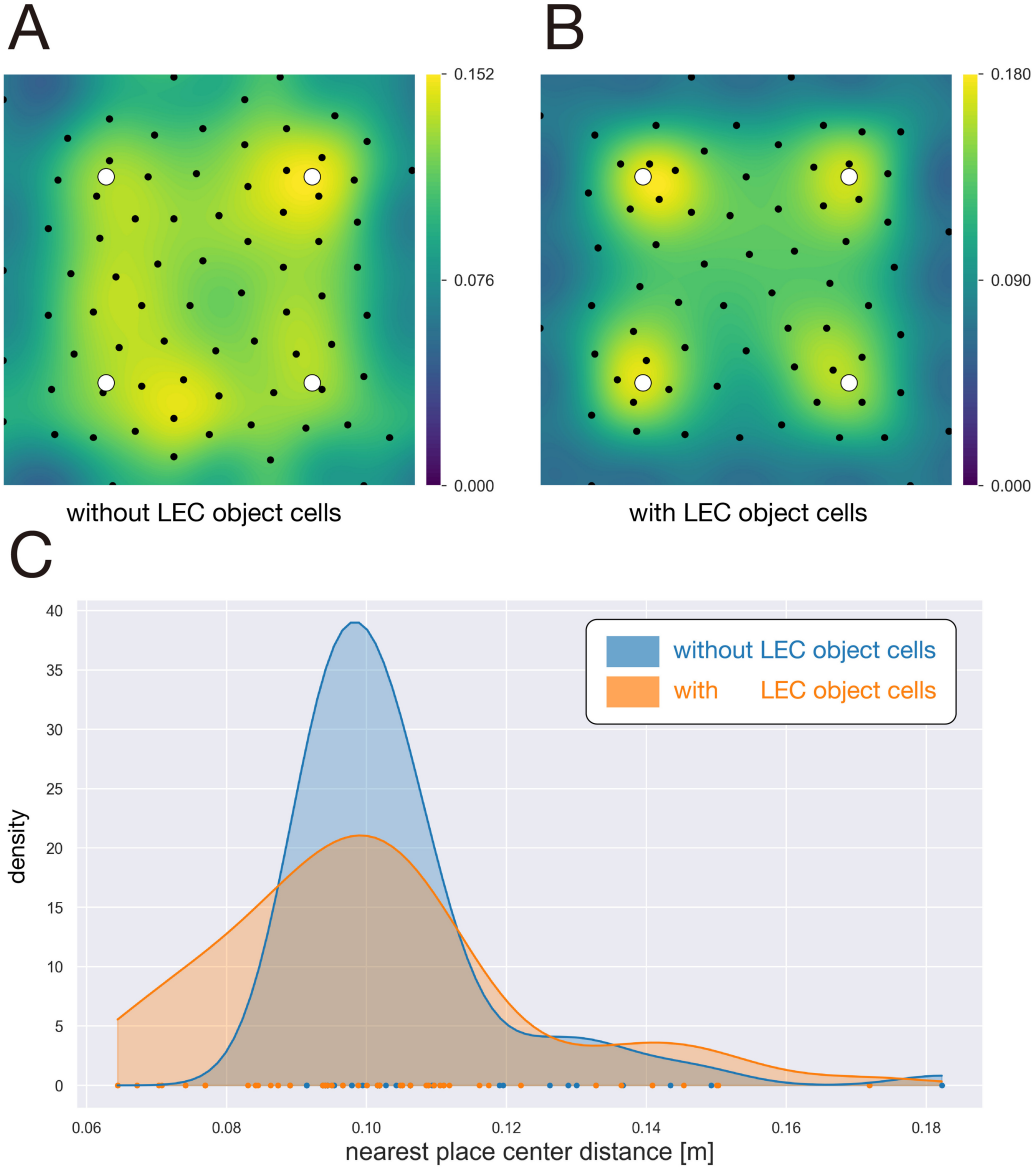
Reproducing the overrepresentation of the place-field centers near the objects. **(A)** Spatial distribution of place-field centers in the sparse coding layer without LEC object cells. Black dots indicate the place-field centers, and the background shows the KDE of their spatial distribution. White dots indicate the objects. **(B)** Spatial distribution of place-field centers in the sparse coding layer with LEC object cells. Black dots indicate the place-field centers, and the background shows the KDE of their spatial distribution. White dots indicate the objects. **(C)** Distribution of the minimum pairwise distances between place-field centers in the sparse coding layer, estimated using KDE. Blue: model without LEC object cells; orange: model with LEC object cells.

When LEC object cells were not included, place fields were distributed uniformly across the environment (Figure 6A). In contrast, when LEC object cells were present, the proportion of place cells with place fields located near objects was markedly increased (Figure 6B), resulting in a clear overrepresentation of proximity to object locations. A similar tendency was also observed in the predictive map layer, indicating that the object-centered overrepresentation produced in the sparse coding layer is preserved in the downstream predictive representation.

To quantitatively assess these differences, we computed the distribution of the minimum pairwise distances between the place-field centers (Figure 6C). In both conditions, the peak of the distribution was located around 10 cm. However, in the presence of LEC object cells, the density at this peak was reduced, while the density in the range of 6–8 cm was increased. This shift indicates a higher local clustering of place fields around objects.

Taken together, these results demonstrate that adding LEC object cells to the model input is sufficient to reproduce the experimentally observed increase in object-related place fields reported by Vandrey et al. (2021). This finding suggests that object-related signals from the LEC can directly modulate hippocampal place representations through their interaction with MEC-derived spatial inputs, providing a mechanistic account of object-induced place field overrepresentation.

### 3.4 Overrepresentation of place cells by LEC reward cells

Gauthier and Tank (2018) recorded hippocampal neuronal activity while mice ran on a virtual linear track with a fixed reward location. They reported that, in addition to conventional place cells, the hippocampus contains neurons that consistently fire near the reward location, independently of spatial remapping. These neurons were interpreted as reward cells distinct from classical place cells. More recently, Issa et al. (2023) recorded neuronal activity in both the MEC and LEC during navigation on a virtual linear track with a fixed reward location. They discovered two distinct populations of reward-related neurons in the LEC: pre-reward cells, which fire before the reward location, and post-reward cells, which fire after the reward location.

**Figure 7:**
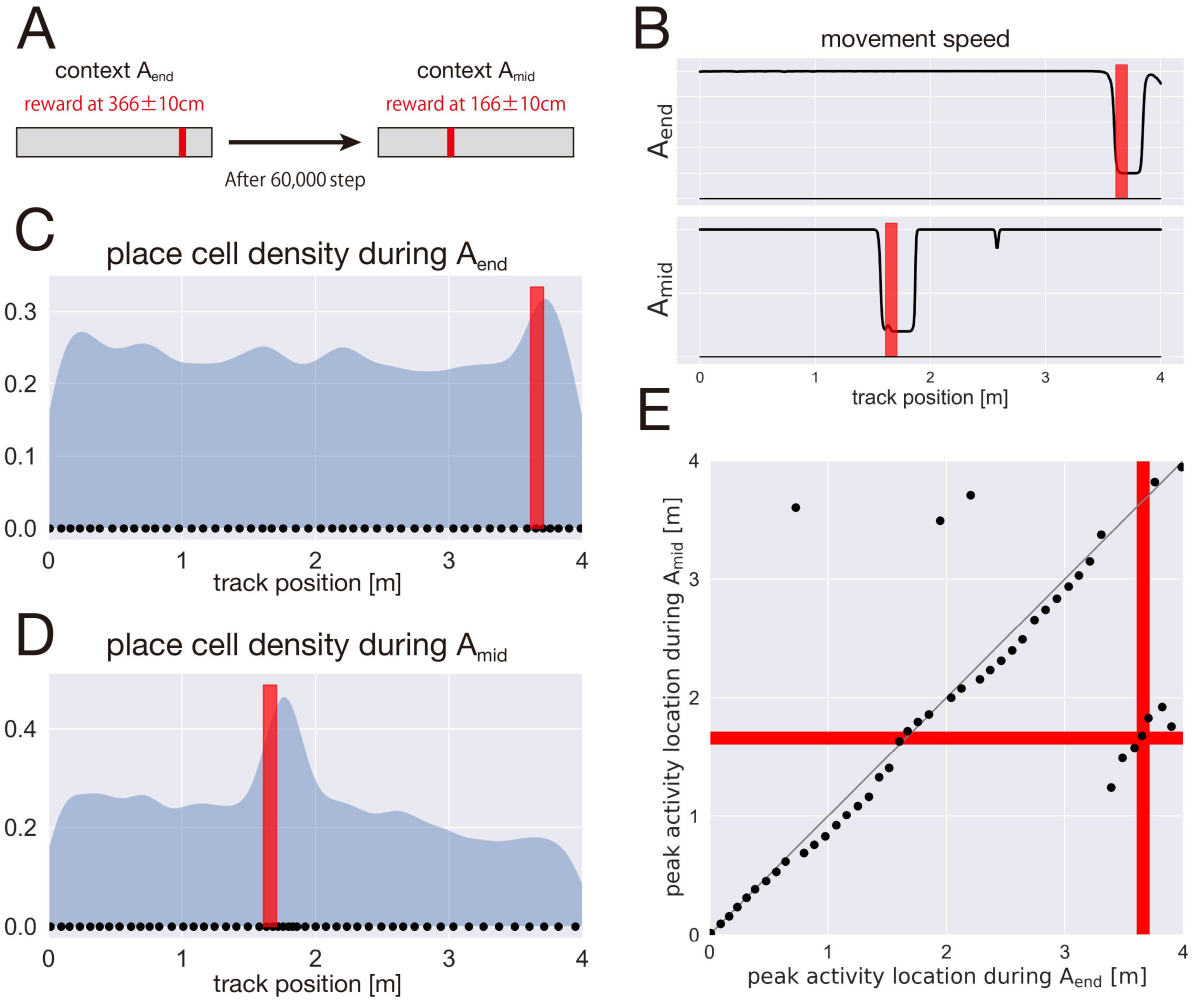
Reproducing the overrepresentation of place-field centers near the reward. **(A)** Experimental setup in a one-dimensional periodic environment (length: 4 m). Red bars indicate the reward zone. During the first 60,000 steps (context *A*_*end*_), reward was delivered within the interval 3.56–3.76 m. Afterward, the reward location was shifted to 1.56–1.76 m for the next 60,000 steps (context *A*_*mid*_). **(B)** Learned policy in each context. Top: policy in context *A*_*end*_. Bottom: policy in context *A*_*mid*_. Red bars indicate the reward zone. **(C)** Spatial distribution of place-field centers in context *A*_*end*_. Black dots indicate place-field centers. **(D)** Spatial distribution of place-field centers in context *A*_*mid*_. Black dots indicate place-field centers. **(E)** Relationship between place-field center locations in context *A*_*end*_ and context A mid. Red bars indicate the reward zone in each context.

In the present study, we hypothesize that the transmission of pre-reward and post-reward signals from the LEC to the hippocampus can account for the overrepresentation of place cells near reward locations. To test this hypothesis, we modeled LEC pre-reward and post-reward cells, and designed a simulation environment inspired by the experimental setup of Gauthier and Tank (2018). Specifically, the agent was placed in a periodic one-dimensional environment of length 4 m. During the first 60,000 steps (context *A*_*end*_), reward was delivered within the interval [3.56 m, 3.76 m]. During the subsequent 60,000 steps (context *A*_*mid*_), the reward location was shifted to [1.56 m, 1.76 m] (Figure 7A).

We first examined the learned policy of the agent. In both context *A*_*end*_ and context *A*_*mid*_, the agent learned to slow down selectively near the reward locations while moving at the maximum speed elsewhere, enabling prolonged stays at the rewarded locations (Figure 7B).

Next, we analyzed the spatial distribution of place cells in each context. In context *A*_*end*_, the place-field density increased around the reward locations at [3.56 m, 3.76 m] (Figure 7C). Similarly, in context *A*_*mid*_, the place-field density increased around the reward locations at [1.56 m, 1.76 m] (Figure 7D).

Finally, we examined how place cells were remapped across contexts (Figure 7E). Consistent with the findings of Gauthier and Tank (2018), we identified neurons that consistently fired near the reward locations despite the change in the reward location. These results indicate that the model reproduces the coexistence of spatially selective place cells and reward-associated neurons observed experimentally, providing a mechanistic link between LEC reward signals and hippocampal place field overrepresentation.

## 4 Discussion

In this study, we proposed a computational model that constructs a predictive map from diverse EC inputs. EC inputs were first transformed into hippocampal place-cell-like representations using NSC, after which a predictive map was learned from these place-cell representations. This framework provides a mechanistic account of how diverse EC inputs can be integrated to form predictive spatial representations in the hippocampus.

Our model suggests a potential correspondence between hippocampal subregions and network components: the sparse coding layer may be associated with CA3, whereas the predictive map layer may correspond to CA1. This interpretation is supported by two observations. First, CA3 is characterized by strong recurrent circuitry, which is consistent with the sparse coding layer. Second, asymmetric place fields with backward skew have been reported predominantly in CA1 rather than CA3 (Mehta et al., 2000; Dong et al., 2021), consistent with the predictive properties observed in the predictive map layer. In the present model, the overrepresentation of place fields near objects or reward locations already emerges in the sparse coding layer and is preserved in the predictive map layer. The sparse coding layer may bind diverse EC inputs to construct a state representation that is suitable for reinforcement learning, while the predictive map layer builds predictive relationships between these states. Such state representations, which overrepresent biologically important locations such as objects or reward sites, may enable more precise estimation of state value around these locations. The predictive map layer therefore provides a mechanism to capture the predictive structure of the environment and support value computation over these state representations. The idea that the hippocampus may construct state representations by integrating diverse EC inputs has also been explored in a recent theoretical study (Bakermans et al., 2025). However, the anatomical circuitry of the hippocampus also includes direct projections from the EC to CA1. In the present model, we did not incorporate direct EC-to-predictive-map connections. Extending the model to include this pathway and investigating its functional role remain important directions for future work.

Classical theories of hippocampal function have interpreted the hippocampus as an associative memory system (Treves and Rolls, 1994). In this framework, hippocampal circuits store and retrieve patterns through recurrent connectivity, particularly in the CA3 region. In contrast, the present study adopts a predictive representation framework, in which hippocampal activity encodes predictive relationships between states rather than simply retrieving stored patterns. Recent computational models of the entorhinal-hippocampal system have explored related ideas from a NeuroAI perspective. For example, the Tolman–Eichenbaum Machine (Whittington et al., 2020) models the hippocampus as a system that supports relational generalization across different tasks and environments. Similarly, the Spatial Memory Pipeline (Uria et al., 2020) describes how spatial representations may be constructed through interactions between sensory inputs and path integration mechanisms. More recently, Chandra et al. (2025) proposed a framework in which spatial representations provide a low-dimensional scaffold for organizing both spatial navigation and more general forms of episodic memory. In this view, memory and behavior can be understood as transitions within a structured representational space. While these approaches differ in their architectural assumptions and goals, they share the broader objective of explaining how the entorhinal-hippocampal system supports predictive or relational representations. In this context, the present model focuses specifically on how predictive maps may be constructed from diverse EC inputs and how such representations interact with reinforcement learning mechanisms.

Previous developmental studies (Langston et al., 2010; Wills et al., 2010) reported that place cells emerge earlier than grid cells in the hippocampal-entorhinal system of preweaning rats. In contrast, our model assumes idealized grid-cell activity as input for generating place-cell representations, which may at first glance appear inconsistent with these experimental findings. However, this apparent discrepancy does not necessarily indicate a conflict with the developmental evidence, because Lian and Burkitt (2021) demonstrated that place-cell-like activity can also be generated from inputs with weak spatial selectivity, rather than from fully developed grid-cell activity. Therefore, our results suggest that robust place-cell representations can emerge from a range of entorhinal input structures during development.

In the predictive map theory of Stachenfeld et al. (2017), grid cells are interpreted as eigenvectors of the SR, suggesting that grid-like activity may emerge from the predictive structure of the environment. In contrast, the present model assumes that grid cells provide upstream inputs from the entorhinal cortex that contribute to the formation of place-cell-like representations through the sparse coding layer. These two interpretations are not necessarily mutually exclusive, because the entorhinal-hippocampal circuit contains strong bidirectional connectivity that could allow predictive representations in the hippocampus to influence entorhinal activity through feedback pathways.

With respect to the specific nature of these entorhinal inputs, we note a limitation regarding the use of boundary-tuned signals within a periodic environment. Since a two-dimensional torus has no physical boundaries by definition, these inputs function as a representational anchor to mitigate numerical discontinuities that arise when the grid spacing is not an exact divisor of the environmental period. Future work should aim to develop more autonomous mechanisms for path integration that can resolve such discontinuities without external coordinate-fixed cues. For instance, employing a continuous attractor network model (Burak and Fiete, 2009) could allow the network dynamics to naturally adapt to the environmental topology. Furthermore, exploring how grid scales are determined—whether they are fixed by a rigid scaling factor (Stensola et al., 2012) or dynamically adjusted to fit the periodic constraints of the environment—remains a critical open question. Such investigations may reveal whether the brain actively aligns its internal grid representation with the global structure of the environment to maintain a seamless spatial map.

A recent experimental study has reported that place fields may shift toward predicted reward locations rather than exhibiting simple backward skew along experienced trajectories (Yaghoubi et al., 2026). Although this phenomenon may appear different from the backward skew predicted by classical predictive map theories, it does not necessarily contradict the predictive map framework. Instead, these findings may reflect the influence of reward prediction signals on hippocampal representations. Dopamine neurons in the ventral tegmental area are well-known to encode reward prediction errors (Schultz et al., 1997). Because dopaminergic signals can influence activity in the entorhinal-hippocampal circuit, reward-related inputs may modulate hippocampal place representations through pathways involving the LEC. Under such a mechanism, place-field shifts could occur toward locations where reward prediction errors are high. Investigating how reward prediction signals interact with predictive spatial representations may therefore help clarify how the hippocampus integrates spatial prediction and value-related information within a unified framework.

Extending this idea, the LEC may encode not only reward-related information but also aversive or punishment-related signals. Anatomical studies have demonstrated excitatory projections from the amygdala to the EC (Sparta et al., 2014), and experimental evidence indicates that neurons in the ventral tegmental area respond not only to rewards but also to aversive stimuli (Brischoux et al., 2009). These pathways suggest a potential route through which punishment-related signals could be conveyed to the entorhinal-hippocampal system. In this framework, the present model could, in principle, generate hippocampal neurons that selectively represent locations associated with aversive experiences. Such an extension would imply that the proposed predictive-map-based mechanism may account not only for place cell activity but also for the formation of memory engrams (Liu et al., 2012) associated with emotionally salient experiences.

## 5 Conclusion

In this study, we proposed a biologically inspired model that constructs predictive maps from the MEC and LEC inputs and supports goal-directed behavior in continuous environments. As the results, the model reproduces experience-dependent place fields called backward skew, object-centered and reward-centered overrepresentation, suggesting a unified computational mechanism linking entorhinal inputs to behaviorally spatial memory.

## Data Availability

The raw data supporting the conclusions of this article will be made available by the authors, without undue reservation.

## References

[B1] Alvernhe A. Save E. Poucet B. (2011). Local remapping of place cell firing in the Tolman detour task. Eur. J. Neurosci. 33, 1696–1705. doi: 10.1111/j.1460-9568.2011.07653.x 21395871

[B2] Bakermans J. J. W. Warren J. Whittington J. C. R. Behrens T. E. J. (2025). Constructing future behavior in the hippocampal formation through composition and replay. Nat. Neurosci. 28, 1061–1072. doi: 10.1038/s41593-025-01908-3 40065185 PMC12081289

[B3] Bennett L. de Cothi W. Muessig L. Rodrigues F. R. Cacucci F. Wills T. J. . (2025). Subicular spatial codes arise from predictive mapping. bioRxiv. [preprint]. doi: 10.2139/ssrn.528451939574744

[B4] Bi G.-Q. Poo M.-M. (1998). Synaptic modifications in cultured hippocampal neurons: dependence on spike timing, synaptic strength, and postsynaptic cell type. J. Neurosci. 18, 10464–10472. doi: 10.1523/JNEUROSCI.18-24-10464.1998 9852584 PMC6793365

[B5] Bono J. Zannone S. Pedrosa V. Clopath C. (2023). Learning predictive cognitive maps with spiking neurons during behavior and replays. eLife 12:e80671. doi: 10.7554/eLife.80671 36927625 PMC10019888

[B6] Bowler J. C. Losonczy A. (2023). Direct cortical inputs to hippocampal area CA1 transmit complementary signals for goal-directed navigation. Neuron 111, 4071–4085.e6. doi: 10.1016/j.neuron.2023.09.013 37816349 PMC11490304

[B7] Brischoux F. Chakraborty S. Brierley D. I. Ungless M. A. (2009). Phasic excitation of dopamine neurons in ventral VTA by noxious stimuli. Proc. Nat. Acad. Sci USA., 106, 4894–4899. doi: 10.1073/pnas.0811507106 19261850 PMC2660746

[B8] Burak Y. Fiete I. R. (2009). Accurate path integration in continuous attractor network models of grid cells. PLoS Comput. Biol. 5, 1–16. doi: 10.1371/journal.pcbi.1000291 PMC2632741 19229307

[B9] Chandra S. Sharma S. Chaudhuri R. Fiete I. (2025). Episodic and associative memory from spatial scaffolds in the hippocampus. Nature 638, 739–751. doi: 10.1038/s41586-024-08392-y 39814883

[B10] Dayan P. (1993). Improving generalization for temporal difference learning: the successor representation. Neural. Comput. 5, 613–624. doi: 10.1162/neco.1993.5.4.613

[B11] de Cothi W. Barry C. (2020). Neurobiological successor features for spatial navigation. Hippocampus 30, 1347–1355. doi: 10.1002/hipo.23246 32584491 PMC8432165

[B12] Deshmukh S. S. Knierim J. J. (2011). Representation of non-spatial and spatial information in the lateral entorhinal cortex. Front. Behav. Neurosci. 5:2011. doi: 10.3389/fnbeh.2011.00069 22065409 PMC3203372

[B13] Dong C. Madar A. D. Sheffield M. E. J. (2021). Distinct place cell dynamics in CA1 and CA3 encode experience in new environments. bioRxiv. [preprint]. doi: 10.1038/s41467-021-23260-3 PMC8137926 34016996

[B14] Fang C. Aronov D. Abbott L. Mackevicius E. L. (2023). Neural learning rules for generating flexible predictions and computing the successor representation. eLife 12:e80680. doi: 10.1101/2020.09.10.292177 36928104 PMC10019889

[B15] Gauthier J. L. Tank D. W. (2018). A dedicated population for reward coding in the hippocampus. Neuron 99, 179–193.e7. doi: 10.1016/j.neuron.2018.06.008 PMC7023678 30008297

[B16] George T. M. de Cothi W. Stachenfeld K. L. Barry C. (2023). Rapid learning of predictive maps with STDP and theta phase precession. eLife 12:e80663. doi: 10.7554/eLife.80663 36927826 PMC10019887

[B17] Georgopoulos A. Kalaska J. Caminiti R. Massey J. (1982). On the relations between the direction of two-dimensional arm movements and cell discharge in primate motor cortex. J. Neurosci. 2, 1527–1537. doi: 10.1523/JNEUROSCI.02-11-01527.1982 7143039 PMC6564361

[B18] Hafting T. Fyhn M. Molden S. Moser M.-B. Moser E. I. (2005). Microstructure of a spatial map in the entorhinal cortex. Nature 436:801–806. doi: 10.1038/nature03721 15965463

[B19] Issa J. B. Radvansky B. A. Xuan F. Dombeck D. A. (2023). Lateral entorhinal cortex subpopulations represent experiential epochs surrounding reward. bioRxiv. [preprint]. doi: 10.1101/2023.10.09.561557 PMC11097142 38272968

[B20] Kuniyoshi Y. Yamazaki T. (2025). “A predictive map model of place cells learned from grid cell activity in continuous spatial environments," in Proceedings of the international conference on neural information processing (ICONIP 2025), Lecture Notes in Computer Science (Auckland: Springer). (to appear). doi: 10.1007/978-981-95-4378-6_28

[B21] Langston R. F. Ainge J. A. Couey J. J. Canto C. B. Bjerknes T. L. Witter M. P. . (2010). Development of the spatial representation system in the rat. Science 328, 1576–1580. doi: 10.1126/science.1188210 20558721

[B22] Lian Y. Burkitt A. N. (2021). Learning an efficient hippocampal place map from entorhinal inputs using non-negative sparse coding. eNeuro 8. doi: 10.1523/ENEURO.0557-20.2021 PMC8266216 34162691

[B23] Liu X. Ramirez S. Pang P. Puryear C. Govindarajan A. Deisseroth K. . (2012). Optogenetic stimulation of hippocampal engram activates fear memory recall. Nature 484, 381–385. doi: 10.1038/nature11028 22441246 PMC3331914

[B24] Mehta M. R. Quirk M. C. Wilson M. A. (2000). Experience-dependent asymmetric shape of hippocampal receptive fields. Neuron 25, 707–715. doi: 10.1016/S0896-6273(00)81072-7 10774737

[B25] O'Keefe J. (1976). Place units in the hippocampus of the freely moving rat. Exp. Neurol. 51, 78–109. doi: 10.1016/0014-4886(76)90055-8 1261644

[B26] O'Keefe J. Dostrovsky J. (1971). The hippocampus as a spatial map. preliminary evidence from unit activity in the freely-moving rat. Brain Res. 34, 171–175. doi: 10.1016/0006-8993(71)90358-1 5124915

[B27] O'Keefe J. Nadel L. (1978). The Hippocampus as a Cognitive Map. Oxford: Clarendon Press.

[B28] O'Keefe J. Recce M. L. (1993). Phase relationship between hippocampal place units and the EEG theta rhythm. Hippocampus 3, 317–330. doi: 10.1002/hipo.450030307 8353611

[B29] Pröll M. Häusler S. Herz A. V. (2018). Grid-cell activity on linear tracks indicates purely translational remapping of 2D firing patterns at movement turning points. J. Neurosci. 38, 7004–7011. doi: 10.1523/JNEUROSCI.0413-18.2018 29976622 PMC6596115

[B30] Rozell C. J. Johnson D. H. Baraniuk R. G. Olshausen B. A. (2008). Sparse coding via thresholding and local competition in neural circuits. Neural Comput. 20, 2526–2563. doi: 10.1162/neco.2008.03-07-486 18439138

[B31] Schultz W. Dayan P. Montague P. R. (1997). A neural substrate of prediction and reward. Science 275, 1593–1599. doi: 10.1126/science.275.5306.1593 9054347

[B32] Solstad T. Boccara C. N. Kropff E. Moser M.-B. Moser E. I. (2008). Representation of geometric borders in the entorhinal cortex. Science 322, 1865–1868. doi: 10.1126/science.1166466 19095945

[B33] Solstad T. Moser E. I. Einevoll G. T. (2006). From grid cells to place cells: a mathematical model. Hippocampus 16, 1026–1031. doi: 10.1002/hipo.20244 17094145

[B34] Sparta D. R. Smithuis J. Stamatakis A. M. Jennings J. H. Kantak P. A. Ung R. L. . (2014). Inhibition of projections from the basolateral amygdala to the entorhinal cortex disrupts the acquisition of contextual fear. Front. Behav. Neurosci. 8:129. doi: 10.3389/fnbeh.2014.00129 24834031 PMC4018552

[B35] Stachenfeld K. L. Botvinick M. M. Gershman S. J. (2017). The hippocampus as a predictive map. Nat. Neurosci. 20, 1643–1653. doi: 10.1038/nn.4650 28967910

[B36] Stensola H. Stensola T. Solstad T. Frøland K. Moser M.-B. Moser E. I. (2012). The entorhinal grid map is discretized. Nature 492, 72–78. doi: 10.1038/nature11649 23222610

[B37] Tanni S. de Cothi W. Barry C. (2022). State transitions in the statistically stable place cell population correspond to rate of perceptual change. Curr. Biol. 32, 3505–3514.e7. doi: 10.1016/j.cub.2022.06.046 35835121 PMC9616721

[B38] Treves A. Rolls E. T. (1994). Computational analysis of the role of the hippocampus in memory. Hippocampus 4, 374–391. doi: 10.1002/hipo.450040319 7842058

[B39] Uria B. Ibarz B. Banino A. Zambaldi V. Kumaran D. Hassabis D. . (2020). The spatial memory pipeline: a model of egocentric to allocentric understanding in mammalian brains. bioRxiv. [preprint]. doi: 10.1101/2020.11.11.378141

[B40] Vandrey B. Duncan S. Ainge J. A. (2021). Object and object-memory representations across the proximodistal axis of ca1. Hippocampus 31, 881–896. doi: 10.1002/hipo.23331 33942429

[B41] Wang C. Chen X. Lee H. Deshmukh S. S. Yoganarasimha D. Savelli F. . (2018). Egocentric coding of external items in the lateral entorhinal cortex. Science 362, 945–949. doi: 10.1126/science.aau4940 30467169 PMC6261310

[B42] Whittington J. C. Muller T. H. Mark S. Chen G. Barry C. Burgess N. . (2020). The tolman-eichenbaum machine: unifying space and relational memory through generalization in the hippocampal formation. Cell 183, 1249–1263.e23. doi: 10.1016/j.cell.2020.10.024 33181068 PMC7707106

[B43] Wills T. J. Cacucci F. Burgess N. O'Keefe J. (2010). Development of the hippocampal cognitive map in preweanling rats. Science 328, 1573–1576. doi: 10.1126/science.1188224 20558720 PMC3543985

[B44] Yaghoubi M. Kumar M. G. Nieto-Posadas A. Mosser C.-A. Gisiger T. Wilson É. . (2026). Predictive coding of reward in the hippocampus. Nature, 651, 414–420. doi: 10.1038/s41586-025-09958-0 41535460

